# Mismatch Negativity Is Not Always Modulated by Lexicality

**DOI:** 10.3389/fnhum.2020.556457

**Published:** 2020-10-30

**Authors:** Stephen Politzer-Ahles, Suyeon Im

**Affiliations:** ^1^Department of Chinese and Bilingual Studies, The Hong Kong Polytechnic University, Hong Kong, Hong Kong; ^2^Hanyang Institute for Phonetics and Cognitive Sciences of Language, Hanyang University, Seoul, South Korea

**Keywords:** mismatch negativity, event-related potentials, Mandarin, lexical access, tone alternation

## Abstract

One factor that is commonly thought to influence MMN amplitude is lexicality; multiple studies have shown that real-word deviants elicit larger MMNs than pseudoword deviants. Here, however, we report data from two experiments challenging this assumption. In the first experiment (*N* = 48), real-word deviants did not elicit more negative MMNs than pseudoword deviants; the acoustic difference between standard and deviant was identical across these comparisons. In this experiment, the pseudoword deviant [pʰa˨˩] differed from a real-word [pʰa˧˥] in tone only; therefore, to test the possibility that the lexicality effect is real but is restricted to pseudowords that differ from real words by at least one segment, we ran a second experiment which included different items and participants, and also included a control comparison in which the pseudoword ([tsʰei˨˩]) differs from all real words by at least one segment (there is no existing Mandarin morpheme pronounced [tsʰei] in any tone). In the second experiment (*N* = 36), both types of pseudowords failed to elicit less negative MMNs than real words. These findings, together with other recent studies failing to show lexicality effects in MMN, challenge the assumption that wordhood reliably influences MMN amplitude.

## Introduction

A key feature of the architecture of language processing is presumably that words of a language, which are represented in the mental lexicon, are processed differently than non-words, which presumably are not. There are many pieces of evidence for this, including the presence of non-productive phonological alternations (i.e., patterns of phonological change that occur in real words but not in non-words; for an example, see Zhang et al., 2011), different patterns of form priming elicited by real-word vs. pseudoword primes (see, e.g., Qiao et al., 2009, for review), and apparently larger N400 components elicited by pseudowords than by words (see, e.g., Friedrich et al., 2006, for review).

The mismatch negativity (MMN) has long been thought to be another aspect of cognition where words and pseudowords pattern differently. The MMN is a component of brain activity traditionally elicited by rarely presented “deviant” stimuli in an oddball design, i.e., where the deviant stimuli are interspersed with frequently presented “standard” stimuli (Näätänen et al., 2007). For example, *da* tends to elicit a more negative frontocentral event-related potential (ERP), about 150–250 ms after presentation, when it is presented as a deviant (e.g., *ba ba ba ba ba ba ba ba* ***da*** *ba ba ba ba* ***da*** *ba*…) than when it is presented as a standard (e.g., ***da da da da da da da da*** *ba* ***da da da da*** *ba* ***da***…). Subtracting the ERP elicited by *da* as a deviant from the ERP elicited by *da* as a standard yields the MMN component.

Crucially, several previous studies have suggested that the MMN is larger (more negative) when the deviant is a real word than when the deviant is a pseudoword. For example, Shtyrov and Pulvermüller (2002) observed a larger MMN elicited by the English real word *bite* (in the context of the standard ^∗^*bipe*) than by the pseudoword ^∗^*pite* (in the context of the standard *pipe*). This lexicality effect on the MMN has been observed in multiple languages and paradigms (see Table 1). A potential explanation for it, per Pulvermüller et al. (2001), is that words are associated with cortical assemblies (Pulvermüller, 1999) which activate when a word is encountered (at least when it is encountered as a deviant; as discussed below, the lexical status of the standard does not appear to influence this effect), whereas pseudowords are not.

**TABLE 1:** Summary of MMN studies comparing words to pseudowords.

Paper	Language	*N*_*participant*_	Pseudoword MMN amplitude^a^	Pseudoword MMN latency^b^	Consistent with lexicality effect	MMN Subtraction^c^
Korpilahti et al. (2001)	Finnish (children)	10	<word^d^	≈word	Yes	Different stimulus, matched
Pulvermüller et al. (2001) exp. 1–2	Finnish	9	<word	≈word	Yes	Different stimulus, matched
Shtyrov and Pulvermüller (2002)	English	10	<word	≈word	Yes	Different stimulus, matched
Sittiprapaporn et al. (2003)	Thai	9	<word	≈word	Yes	Unclear^e^
Pulvermüller et al. (2004)	Finnish	9	<word	>word	Yes	Different stimulus, matched
Pettigrew et al. (2004a)	English	30^f^	≈word	≈word	No	Same stimulus
Pettigrew et al. (2004b)	English	10	≈word^g^	≈word	Arguably no^g^	Same stimulus
Endrass et al. (2004)	German	17	<word^h^	≈word	Yes^h^	Same stimulus
Ylinen et al. (2009)	Finnish	10	≈word	<word	No^i^	Different stimulus, unmatched
Zora et al. (2015)	English	11	≈word	≈word	No	Different stimulus, matched
Honbolygó (2019)	Hungarian	Not reported	≈word	≈word	No	Same stimulus

*^a^By “<” here, we mean that the MMN is smaller (i.e., more positive) for pseudowords than it is for words. ^b^In some of these cases, statistical tests for MMN latency are not presented in the paper, and thus our entry here is based on our own visual inspection of the grand average waveforms and should not imply that there are statistically significant differences. ^c^“Different stimulus, matched”: the standard, which is different from the deviant, is subtracted from the deviant. The acoustic difference between standard and deviant is identical across the word blocks and pseudoword blocks. “Different stimulus, unmatched”: the standard, which is different from the deviant, is subtracted from the deviant, and the acoustic difference between standard and deviant is not identical across blocks. “Same stimulus”: ERPs to a stimulus elicited as a standard are subtracted from ERPs to the same stimulus elicited as a deviant. ^d^In a late time window, but not an early time window. ^e^The paper says “MMN was obtained by subtracting the response to the standard from that to the deviant.” This could mean subtracting the response to the standard in a given block from that to the deviant in a given block (this would be a “different stimulus, unmatched” subtraction paradigm) or it could mean subtracting the response to a given sound presented as a standard in one block from that to the same sound presented as a deviant in another block (this would be a “same stimulus” subtraction paradigm). ^f^Across two groups (N = 15 each) with different stimulus-onset asynchronies. ^g^See their Figure 4. There is another comparison in which the pseudoword deviants appear to elicit a smaller (more positive) MMN than real-word deviants (their Figure 5), but in this comparison the word-pseudoword comparison is confounded with a directional asymmetry: the conditions involving a transition from a word standard to pseudoword deviant also involve a transition from a monophthong standard to a diphthong deviant, whereas the conditions involving a transition from a pseudoword standard to a word deviant are the opposite (diphthong standard to monophthong deviant). There are many reasons to suspect that moving from a less complex standard to a more complex deviant may elicit a bigger MMN than vice versa (see, e.g., Politzer-Ahles et al., 2016, for review). ^h^As in Pettigrew et al. (2004b), the word-pseudoword comparison here is confounded with a directional asymmetry. Here, however, it is not as obvious to us whether this confound provides an alternative account for the apparent lexicality effect. ^i^The design of this study is complicated, and the experiment was not designed to test lexicality effects. In order to hold stress pattern constant, for the purpose of this table we are comparing the “pseudowords with unfamiliar stress pattern” to the “words with unfamiliar stress pattern”.*

The present study was designed to take advantage of this lexicality effect on the MMN to examine the mental representation of phonologically derived forms that do not exist as citation forms (i.e., the canonical form of a word, such as what would be listed in a dictionary) in their language but which are still commonly used (see “Materials and Methods” section for details). In the course of that experiment (Experiment 1 of the present paper), however, we found that the widely reported effect of lexicality on MMN amplitude did not replicate. Furthermore, it turns out that, despite the somewhat widely held assumption that words elicit larger MMNs than pseudowords, the most recent MMN studies to include words and pseudowords generally failed to show this effect (see Table 1; most of these studies, however, were not designed to examine the lexicality effect in particular). We thus ran a second experiment in attempt to replicate and extend our failure to replicate the lexicality effect. In both experiments, real words failed to elicit larger MMNs than fairly word-like pseudowords; in our second experiment, however, a potential lexicality effect was observed when using less word-like pseudowords.

All materials, analysis code, data, and participant demographic information for the experiments is available at https://osf.io/f4rnw/. All research was approved by the Human Subjects Ethics Sub-committee at the Hong Kong Polytechnic University (#HSEARS20160918002).

## Experiment 1

### Experiment Design and Rationale

The critical part of the experiment included two types of blocks, as listed below:

1. **Real word vs. pseudoword comparison**
  a. **Real-word deviant:** [kʰai˨˩] [kʰai˨˩] [kʰai˨˩] … **[k**ʰ**a˨˩]**
  b. **Pseudoword deviant:** [pʰai˨˩] [pʰai˨˩] [pʰai˨˩] … **[p**ʰ**a˨˩]**

The deviant [kʰa˨˩] is the pronunciation of the existing Mandarin morpheme 卡 (“card” or “stuck”), and it is interspersed with standards [kʰai˨˩], which is the pronunciation of several existing low-frequency morphemes (凯、慨、恺), two of which are mainly used in adapting foreign names. On the other hand, the deviant [pʰa˨˩] does not correspond to any existing morpheme in Mandarin; neither does the standard [pʰai˨˩].

The experiment included two additional types of blocks, which were the initial focus of the experiment design:

2. **Real word vs. surface allomorph comparison**
  a. **Real-word deviant:** [pʰai˧˥] [pʰai˧˥] [pʰai˧˥] … **[p**ʰ**a˧˥]**
  b. **Surface allomorph deviant:** [kʰai˧˥] [kʰai˧˥] [kʰai˧˥] … **[k**ʰ**a˧˥]**

The deviant [pʰa˧˥] is the pronunciation of several real morphemes (e.g., 爬, “climb”), as is the standard [pʰai˧˥] (e.g., 牌, “card”). Neither [kʰa˧˥] nor [kʰai˧˥] is the pronunciation of the citation form of any Mandarin morpheme. Whether they count as existing morphemes, however, is unclear, because of complications caused by phonological alternations, as described immediately below.

Mandarin Chinese has a tone sandhi pattern which causes low-tone syllables to change to rising-tone syllables under certain circumstances. This pattern, combined with accidental gaps in the language, leads to some syllables with ambiguous lexical status. For example, [kʰa˧˥] does not exist as a citation form in Mandarin (it does not occur, for example, in dictionaries; and participants in the present study reported that they do not know any characters with this pronunciation). [kʰa˨˩], however, does; and as it is a syllable with low tone, it is sometimes pronounced with rising tone as a result of tone sandhi, e.g., in the following example:

**Table:** 

Orthography:	我	电脑	卡	死了！
Underlying:	wo˨˩	tjɛn˥˩nɑu˨˩	**k**ʰ**a**˨˩	sɹ̩˨˩ lɤ
Surface:	wo˨˩	tjɛn˥˩nɑu˨˩	**k**ʰ**a**˧˥	sɹ̩˨˩ lɤ
Gloss:	I/me	computer	slow	INTENSIFIER
Literal: “My computer is slow as heck!”				

[Note that some researchers argue that the rising tone that is produced as a sandhi-derived variant of an underlying low tone is not the “same” phonological tone as the true rising tone; see, e.g., Yuan and Chen, 2015. This is motivated in part by the fact that these tones are incompletely neutralized – i.e., a word that’s pronounced with rising tone because of tone sandhi has a slightly different F0 contour than a word that’s pronounced with rising tone because that’s its underlying tone (Peng, 2000). It is unclear whether or not listeners are sensitive to this slight difference (Politzer-Ahles et al., 2019). In any case, the fact that two forms have gradient phonetic differences does not entail that they have different phonological forms (Durvasula and Du, 2020). Importantly, in the present study, the stimuli were digitally manipulated to ensure identical F0 contours (see the section “Materials and Methods”) and thus this difference cannot explain our results. Our study design and predictions do not make any assumptions about whether the rising tone on a sandhi-derived syllable like [kʰa˧˥] is the same or different from an underlyingly rising tone.]

It is unclear, therefore, whether such syllables (accidental gaps which can be pronounced as a result of tone sandhi) would be processed like pseudowords (since native speakers generally do not consider them to be existing words of the language) or like real words (since they are frequently used and heard in connection with meaningful messages, and thus per the account of Pulvermüller et al. (2001) they would form cortical assemblies). The initial goal of the experiment was to use the comparison between these “surface allomorph” deviants^1^ and the matched real-word deviants in order to test whether these forms are treated as real words or pseudowords. On the other hand, the first comparison listed above (between real words and uncontroversial pseudowords) was only intended as a manipulation check, as it was expected to show the lexicality effect widely assumed to occur in the MMN.

The experiment thus had a 2 × 2 design. In the real word vs. pseudoword comparison (the control or manipulation check comparison), we expected larger (more negative) MMNs for the real words than for the pseudowords. In the real word vs. surface allomorph comparison, we expected no difference if the surface allomorphs were treated as real words, but expected larger (more negative) MMNs for the real words if the surface allomorphs were treated as pseudowords.

In this experiment, we use different stimuli for standards (e.g., [kʰai˨˩]) than we do for deviants (e.g., [kʰa˨˩]). This is counter to what is often considered best practice in MMN research with language stimuli: using the same stimuli as deviants in some blocks and standards in other blocks, which allows the standard ERP to be subtracted from the deviant ERP elicited by the exact same physical stimulus. The latter method provides the best means for accounting for endogenous ERP effects triggered by physical properties of the stimulus: any weird ERP patterns caused solely by physical properties of the diphthong [ai], for example, would be present in both the standards and the deviants, and thus would be subtracted out of the eventual MMN waveforms. A limitation of that paradigm, however, is that it yields MMN asymmetries: it would entail, for example, having [a] deviants among [ai] standards in one block, and [ai] deviants among [a] standards in another block, and we know that going from a more simple (monophthong) standard to a more complex (diphthong) deviant elicits larger MMNs than the converse (Timm et al., 2011, among others). Likewise, going from rising-tone standards to low-tone deviants would elicit larger MMNs than going from low-tone standards to rising-tone deviants (Li and Chen, 2015; Politzer-Ahles et al., 2016). Thus, we chose instead to follow the paradigm of Shtyrov and Pulvermüller (2002), which ensures that the physical contrast between standard and deviant is held constant across the blocks to be compared. For example, in every block in our experiment, the physical difference between standard and deviant is always a difference between [ai] rimes (standards) and [a] rimes (deviants).

One final potential concern with this design is that not only the deviants, but also the standards, differ in lexical status. The findings of Shtyrov and Pulvermüller (2002) suggest that only lexical status of the deviant, and not lexical status of the standards, modulates the MMN. In fact, according to the account of MMN lexical effects put forth by Pulvermüller et al. (2001), the lexicality effect observed in the MMN has nothing to do with the processes generating the MMN itself. Rather, it is totally due to processes that occur in the processing of individual words (hearing a real word activates a cortical assembly, whereas hearing a pseudoword does not), not processes related to the detection of differences. The use of the MMN paradigm is just a convenient way to subtract out physical differences between different words, and thus to argue that larger ERPs elicited by, e.g., *pipe* as opposed to *bipe* are due to their lexical status and not to low-level differences in the processing of *p* onsets as opposed to *b* onsets. For this reason, we assumed that differences in the lexical status of the standards in our experiment should not affect our results. Even if they did affect our results, though, the most likely way they would do so would be by triggering larger MMNs when the standard and deviant differ in lexicality [as noted above, however, Shtyrov and Pulvermüller (2002) did not observe such an effect]. If this pattern happens, it would not bias us against observing a lexicality effect. For our pseudoword deviant, the standards are also pseudowords. For our real-word deviant meant to be compared with the pseudoword deviant, the standards are real words but are less common (they are mainly used in borrowings). Thus, if anything, the lexical difference between standard and deviant in the real-word deviant block is bigger than that in the pseudoword deviant block; in other words, if our design biases us, it biases us *toward* observing a lexicality effect.

### Methods

#### Participants

Forty-eight right-handed native speakers of Mandarin (40 women and 8 men, 19–36 years old [mean: 23]) participated in the experiment. This target sample size was chosen based on a simulation-based power analysis^2^ suggesting that a sample of 48 participants would give an 80% chance to detect a −1 μV effect (based on previous pilot data). An additional one participant took part in the experiment but their data were corrupted by environmental noise present for the entire experiment and thus were not included in the analysis. All participants provided informed consent and were compensated for their participation.

#### Materials

Multiple tokens of each of the eight syllables in the experiment design were produced by a male native Mandarin speaker from Beijing.

From these, clear tokens of the consonants [kʰ] an [pʰ] were excised, as were clear tokens of the four vowels [ai˧˥], [a˧˥], [ai˨˩], and [a˨˩]. Each consonant was spliced to each vowel, yielding eight stimuli; the stimuli were then intensity-normalized to 75 dB. This procedure was repeated five times to yield 40 stimuli (five tokens of each word).

This procedure ensured that the physical difference between standards and deviants (i.e., the difference between [ai] and [a] rimes) was identical across all conditions. Furthermore, the difference between conditions within a comparison was also identical. For instance, in the real-word vs. pseudoword comparison, the exact same rime tokens are used in both the blocks with real-word deviants and the blocks with pseudoword deviants.

#### Participant Screening

All participants were pre-tested to ensure that they recognized the real words and did not consider any of the pseudowords to be real words. They were given a list of syllables written in Hanyu Pinyin (the Romanization system used for Mandarin within China) and instructed to write as many Chinese characters as they can think of that had each pronunciation, without checking a dictionary; they were told they can leave an entry blank if they do not know any characters corresponding to that Hanyu Pinyin syllable. Participants were only registered for the experiment if they left blank all the syllables that we treat as pseudowords in this experiment, and if they wrote down at least one Chinese character for each of the syllables that we treat as real words in this experiment.

#### Procedure

Stimulus presentation and output of event markers to the EEG acquisition system was handled by Presentation (Neurobehavioral Systems, Inc.). Stimuli were presented binaurally through tube earphones (Etymotic Research). Participants watched self-selected videos from Netflix or online video-sharing platforms, with subtitles on and sound off, while passively listening to the experimental stimuli. The experiment consisted of twelve blocks (three per oddball condition), presented in a different random order for each participant. Each block began with a series of 20 standards, followed by a pseudorandom series of 50 deviants and 290 standards arranged such that each deviant was preceded by 2–10 standards. Thus, deviants made up 14.7% of a block after the initial sequence of 20 standards, or 13.9% of an entire block. Every time a standard or deviant was to be presented, it was randomly selected from one of the five tokens of that item. The inter-trial interval varied randomly from 500 to 520 ms. After each block, the participant was able to take a break for as long as they wanted before beginning the next block. The entire experimental session, including setup and debriefing, lasted around 2–2.5 h.

#### EEG Acquisition

The continuous EEG was recorded using a SynAmps 2 amplifier (NeuroScan, Charlotte, NC, United States) connected to a 64-channel Ag/AgCl electrode cap. The channels followed the 10–20 system. Polygraphic electrodes were placed above and below the left eye (forming a bipolar channel to monitor vertical EOG), at the left and right outer canthi (forming a bipolar channel for horizontal EOG), and at the right and left mastoids (to be digitally averaged offline for referencing). A channel located halfway between Cz and CPz served as the reference during the recording and AFz as the ground. Impedances were kept below 5 kΩ. The EEG was digitized at a rate of 1000 Hz with an analog bandpass filter of 0.03–100 Hz. A Cedrus Stimtracker interfaced between the experiment control software and the EEG acquisition software; in addition to recording event markers sent from Presentation, it also recorded the onset and offset of the audio stimuli via an auditory channel, but these markers were not used in the present analysis.

#### Data Processing and Analysis

For each participant, data were imported to EEGLAB (Delorme and Makeig, 2004) and, if necessary, up to three bad channels were interpolated. All scalp channels were re-referenced to the average of both mastoids. The data were then segmented into epochs from 200 ms before to 700 ms after each vowel onset. The data were then decomposed into as many independent components as there were channels (minus the mastoids and any bad channels). For each participant, up to two components associated with blinks or saccades were identified based on manual inspection and removed. The data were then subjected to a 30 Hz low-pass filter and baseline-corrected using the 100-ms pre-stimulus interval, and epochs with artifact remaining were marked for removal based on an amplitude threshold (trials with amplitudes exceeding | 75| μV in the interval from 150 ms before to 600 ms after the stimulus onset were removed). The number of trials remaining per condition per participant is available in the spreadsheet at https://osf.io/f4rnw/.

The first deviant of every block was removed from analysis, and ERPs elicited by the deviants that remained in the data after this procedure and artifact rejection were averaged within each condition for each participant. For standards, all standards before the first deviant in a block were removed, and all standards that immediately followed a deviant were removed; standards remaining in the data after this procedure and artifact rejection were averaged within each condition for each participant. MMNs were calculated for each participant by subtracting the averaged standard waveform from the averaged deviant waveform from the corresponding condition.

Statistical analysis was carried out using cluster-based permutation tests (Maris and Oostenveld, 2007) in the FieldTrip package of MATLAB scripts (Oostenveld et al., 2011). This procedure allows a single test over the entire electrode array and the entire epoch (or any theory-motivated subset of electrodes and/or time points) to detect the presence of ERP differences between conditions, while controlling for the multiple comparisons problem if the analysis were performed over multiple tests on discrete time windows and/or channel selections. For each datapoint (each sample at each channel) from 0 to 600 ms post-stimulus-onset, the amplitude of the MMN elicited by the real-word deviant was compared to the amplitude of the MMN elicited by the corresponding pseudoword or surface allomorph deviant using a one-tailed *t*-test. Clusters of spatiotemporally adjacent data points with significant differences were identified (to be included in a cluster, a data point needed to have an uncorrected *p* less than 0.05, and at least two spatially adjacent samples from the same time point which also had *p* < 0.05), and the *t* statistics from all samples within a given cluster were summed to yield a test statistic. The data were then permuted 5000 times and the abovementioned clustering procedure was repeated for each permutation. The proportion of permutations which yielded a larger test statistic than the observed data was the *p*-value for the test.

### Results

**FIGURE 1:**
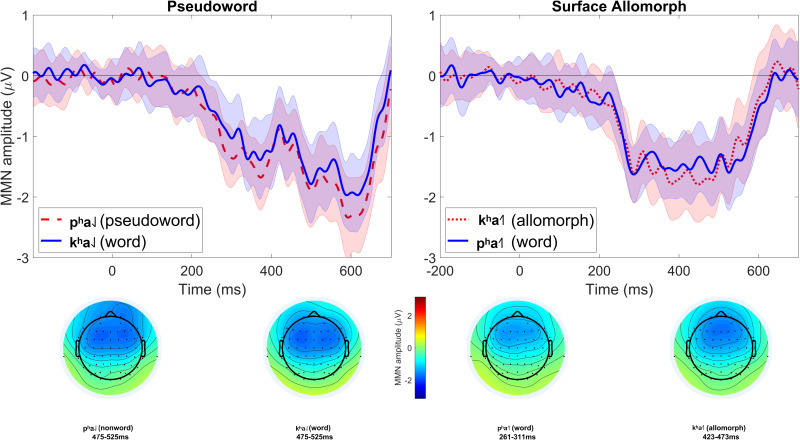
MMNs elicited in Experiment 1. Each subplot compares the MMN (at electrode Fz) elicited by one critical stimulus type (pseudowords, surface allomorphs) to that elicited by corresponding real-word stimuli (i.e., the subplot labeled “Pseudoword” compares the MMN elicited by pseudoword deviants to that elicited by real-word deviants). Each MMN is the result of subtracting ERPs elicited by standards from the ERPs elicited by corresponding deviants in the same block. The shaded ribbon around each MMN wave is a difference-adjusted Cousineau–Morey interval (Cousineau, 2005; Morey, 2008); at any given time point, if one MMN’s interval does not include the other MMN’s mean and vice versa, the two MMNs are likely to be significantly different from one another at that time point. The bottom portion of the figure shows topographic plots of each MMN from a 51-ms time window centered on that MMN’s most negative point.

**FIGURE 2:**
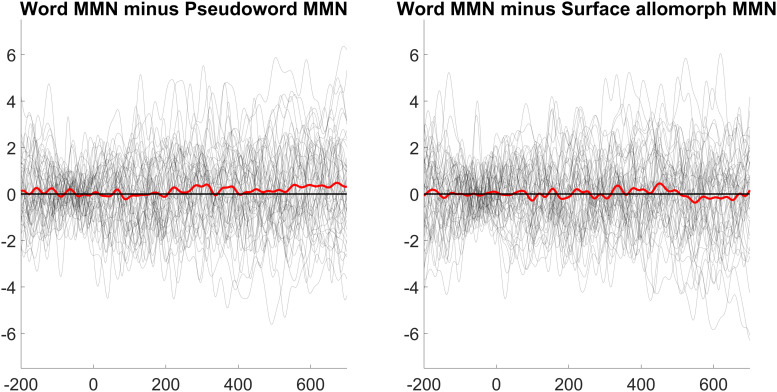
Lexicality effects (MMN elicited by word deviants minus MMN elicited by pseudoword or surface allomorph deviants) for each participant (gray lines), as well as the grand average of these effects (thick red line), at electrode Fz.

The lowest number of trials remaining in a single cell was 83 (56%). MMNs (at electrode Fz) for each condition are shown in Figure 1. Lexicality difference waves (real-word MMN minus pseudoword or surface-allomorph MMN) for each participant are shown in Figure 2. Supplementary Figures showing ERPs at all scalp channels are available at https://osf.io/f4rnw/. Highly significant MMNs (i.e., significant differences between deviants and corresponding standards) were observed in each condition; *p*s < 0.001. It is clear from these figures, however, that real words did not elicit more negative MMNs than either uncontroversial pseudowords or surface allomorphs. Statistical analysis (summarized in Table 2) confirmed this impression.

**TABLE 2:** Summary of Experiment 1 results.

Comparison	Real-word deviant	Other deviant	MMN for real-word deviant	MMN for other deviant	Real word MMN vs. other MMN
Pseudoword vs. real word	[kʰa˨˩]	[pʰa˨˩]	131–600 ms, *p* < 0.001	202–600 ms, *p* < 0.001	Real≈Pseudo, *p* = 0.547
Allomorph vs. real word	[pʰa˧˥]	[kʰa˧˥]	165–600 ms, *p* < 0.001	191–600 ms, *p* < 0.001	Real≈Allomorph, *p* = 0.685

*“Other” refers to whichever condition is not the real-word condition in that comparison (e.g., in the comparison between real words and pseudowords, the “other” deviant is the pseudoword deviant). The columns “MMN for real-word deviant” and “MMN for other deviant” report the p-value for the one-tailed test comparing the deviant to the corresponding standard; the column “Real word MMN vs. other MMN” reports the p-value for the one-tailed test comparing the real-word deviant’s MMN to the other deviant’s MMN.*

In the manipulation-check comparison between real words and pseudowords, real words did not elicit significantly more negative MMNs than pseudowords (*p* = 0.547). For exploratory analysis, we tried repeating this analysis with different sets of clustering settings for the permutation test (e.g., using a more stringent *p*-value threshold for cluster formation, which makes the test more sensitive to focal effects, and/or adjusting the minimum number of neighboring channels needed for a data point to be included in a cluster), but these different versions of the test still did not detect a significant difference between the two MMNs.

As this manipulation check did not show a significant lexicality effect, the results from the comparison between real words and surface allomorphs is probably not interpretable. For thoroughness, however, we tested this comparison as well; once again, real words did not elicit significantly more negative MMNs than surface allomorphs (*p* = 0.685). This result cannot be treated as evidence that surface allomorphs were processed as real words, however, since the result from the manipulation check suggests that this manipulation does not effectively distinguish real-word from pseudoword processing anyway.

### Discussion

Contrary to our expectation, pseudowords did not yield smaller MMNs than real words. This surprising result suggests that either (1) words do not actually reliably elicit bigger MMNs than non-words; (2) words do elicit bigger MMNs than non-words and our results are a Type II error or a result of a flaw in our experimental design; or (3) there is something special about the stimuli we used, or about Mandarin in general, which makes it behave differently than other language or other types of words.

Experiment 2 is a near replication of the previous experiment, using new participants and new stimuli in order to increase the generality of results (as one of the limitations of the MMN paradigm is that a single experiment usually only includes a small number of items). If the second experiment again fails to yield a significant lexicality effect, this will provide evidence against the possibility that the lack of lexicality effect was due to a Type II error or an idiosyncracy of the particular tokens used. In Experiment 2, we also test another type of pseudoword, to help further test the possibility that the lack of lexicality effect was due to the type of Mandarin pseudowords we used.

## Experiment 2

The pseudoword used in Experiment 1, [pʰa˨˩], differs from real words only in terms of its tone. That is to say, there are other real words in Mandarin which have the same segments but a different tone (e.g., [pʰa˧˥] 爬 “climb”). Could this have made the stimuli more word-like than the pseudowords used in most other MMN studies examining lexicality?

From a purely phonological perspective, there should be nothing special about a pseudoword that differs from real words because of tone. A tone is a phoneme (see, however, Zhang, 2010, for complications in the phonological analysis of tone), and a pseudoword like [pʰa˨˩] is a neighbor of a real word (i.e., the pseudoword differs from the real word only in terms of addition, removal, or replacement of one phoneme). This is no different than the way that a typical English pseudoword, like “bipe,” is a neighbor (differing by one phoneme) of a real word “pipe.” However, the psychological status of tone, and the way it is used in lexical access, may be different than the psychological status of other phonological cues (see, e.g., O’Seaghdha et al., 2010). Wiener and Turnbull (2013), for example, provide evidence that Mandarin pseudowords which differ from real words by just the tone are harder to recognize as pseudowords, compared to pseudowords that differ from real words by just one segment. It is possible, then, that the pseudowords in the previous experiment were more word-like than pseudowords used in most other MMN experiments on lexicality.

Therefore, in the current experiment we also include another pair of conditions, with the pseudoword deviant [tsʰei˨˩]; the segmental syllable [tsʰei] does not occur in any tone in Mandarin but also does not violate any strong phonological constraints, and thus is arguably more comparable to the pseudowords used in other studies (such as English “bipe”).

The experiment also included the same conditions as Experiment 1 (albeit realized with different items this time). It thus had a 2 × 3 design with the following conditions.

1. **Real word vs. difficult (more word-like)pseudoword comparison**
  a. **Real-word deviant:** [pan˨˩] [pan˨˩] [pan˨˩] … **[pən˨˩]**
  b. **Pseudoword deviant:** [man˨˩] [man˨˩] [man˨˩] … **[mən˨˩]**
2. **Real word vs. easy (less word-like)pseudoword comparison**
  a. **Real-word deviant:** [tai˨˩] [tai˨˩] [tai˨˩] … **[tei˨˩]**
  b. **Pseudoword deviant:** [tsʰai˨˩] [tsʰai˨˩] [tsʰai˨˩] … **[ts**ʰ**ei˨˩]**
3. **Real word vs. surface allomorph comparison**
  a. **Real-word deviant:** [man˧˥] [man˧˥] [man˧˥] … **[mən˧˥]**
  b. **Surface allomorph deviant:** [pan˧˥] [pan˧˥] [pan˧˥] … **[pən˧˥]**

For the first comparison, which is a near-replication of the critical comparison from Experiment 1, the real-word deviant is [pən˨˩] (本, a classifier for books), presented along with standards [pan˨˩], which is also the pronunciation of an existing morpheme (版, “edition”). The pseudoword [mən˨˩] is interspersed with standards [man˨˩], which is the pronunciation of an existing morpheme (滿, “full”). As discussed with regards to Experiment 1, however, previous evidence suggests that the lexicality of the standard does not influence the MMN (Shtyrov and Pulvermüller, 2002).

For the second comparison, between real words and easy (i.e., easy to identify as a pseudoword) pseudowords, the real word deviant [tei˨˩] (得, “must”) is interspersed with standards [tai˨˩], which is the pronunciation of some low-frequency existing morphemes (e.g., 歹, “evil”). The easy (i.e., easy to identify as a pseudoword) pseudoword deviant [tsʰei˨˩] is interspersed with standards [tsʰai˨˩], which is the pronunciation of an existing morpheme (彩, “color”).

The last comparison is between real words and surface allomorphs; while this comparison is no longer of primary experimental interest (given that it is no longer clear that this paradigm is useful for measuring the lexical status of the surface allomorph), it is included to allow comparison with Experiment 1. The real word deviant [mən˧˥] (门, “door”) is interspersed with standards [man˧˥], which is the pronunciation of several existing morphemes (e.g., 蛮, an intensifier). The surface allomorph deviant [pən˧˥], which is a potential derived pronunciation of 本 (a classifier for books) is interspersed with standards [pan˧˥], which is the pronunciation of an existing morpheme (盘, “plate”).

Note that the purpose of these experiments is not to examine the processes involved in recognizing the difference between different tones, or the processes involved in recognizing the difference between vowels. Rather, we are just using these differences to elicit MMNs, because MMNs have been shown in previous research to be a convenient way to compare words and pseudowords while subtracting out the endogenous ERP responses related to low-level acoustic properties of the stimuli which may be different between words and pseudowords. Thus, the fact that Experiment 1 examines a vowel contrast and Experiment 2 examines a tone contrast is not important to our predictions, given that the experiment is not about the contrasts themselves; it is merely using these contrasts to elicit MMN difference waves. As we have ensured that the magnitude of the standard-deviant contrast is identical across the word and pseudoword conditions, we have no *a priori* reason to expect that the nature of the contrast used to elicit MMNs would interact with lexical status of the stimuli.

### Methods

#### Participants

Thirty-six right-handed native speakers of Mandarin (26 women and 10 men, 19–35 years old [mean: 23]) participated in the experiment. The sample was smaller than that of the previous experiment because we became unable to collect data for the last several months of the project (our lab is located in the Hong Kong Polytechnic University, which was the site of violent clashes between police and protesters in November 2019 and was mostly closed for repairs in November 2019 to January 2020, and was then mostly closed during the COVID-19 pandemic through to the time of this writing, April 2020). An additional eleven participants took part in the experiment but were not included in the analysis because either they did not pass the screening (*N* = 5), which was done after data collection, rather than before, for this experiment, or because their data were corrupted by excessive artifacts that ICA decomposition did not ameliorate (*N* = 6). All participants provided informed consent and were compensated for their participation.

#### Materials

Stimuli were generated in the same way as in Experiment 1, except that they were spoken by a female native Mandarin speaker.

#### Participant Screening

Participants were screened in the same way as in Experiment 1, except that instead of screening them before allowing them to register for the experiment, we instead ran all volunteers in the experiment and conducted the screening test after the EEG session. Participants who completed the EEG recording but whose responses in the screening test did not meet our criteria for inclusion were removed from subsequent analyses.

#### Procedure

Stimulus presentation and output of event markers to the EEG acquisition system was handled by Presentation (Neurobehavioral Systems, Inc.). The experiment consisted of fourteen blocks (two per oddball condition, plus two “control” blocks; see below), presented in a different random order for each participant. Each block began with a series of 20 standards, followed by a pseudorandom series of 75 deviants and 437 standards arranged such that each deviant was preceded by 2–10 standards. Thus, deviants made up 14.6% of a block after the initial sequence of 20 standards, or 14.1% of an entire block. Every time a standard or deviant was to be presented, it was randomly selected from one of the five tokens of that item. The inter-trial interval varied randomly from 500 to 520 ms. After each block, the participant was able to take a break for as long as they wanted before beginning the next block. The entire experimental session, including setup and debriefing, lasted around 3–3.5 h.

In addition to the oddball blocks, the experiment included two “control” blocks, following the paradigm of Schröger and Wolff (1996). In this block, each of the deviants from the other blocks appears with 14.3% (i.e., 1 out of 7 trials) frequency. This was accomplished by including each of the six deviants, plus one additional pseudoword item [tʰei˧˥], and presenting these in a fully random order. As discussed by Schröger and Wolff (1996), subtracting ERPs elicited by these items, rather than subtracting ERPs elicited by standards, is a more effective way to isolate the MMN from the N1 component.

#### EEG Acquisition

The acquisition setup and parameters were identical to that of Experiment 1.

#### Data Processing and Analysis

Data processing and statistical analysis was identical to that in Experiment 1, except that MMNs were calculated by subtracting the ERPs elicited by the control version of each stimulus (the stimulus when presented in the control block, where there were no standards or deviants), rather than the standard version of each stimulus, from the ERPs elicited by the deviants (The results obtained when calculated MMNs by subtracting the standards are qualitatively similar to those obtained with this method, except in the case of the easy pseudoword comparison—this case is pointed out in the “Results” section below). The number of trials remaining per condition per participant is available in the spreadsheet at https://osf.io/f4rnw/.

### Results

**FIGURE 3:**
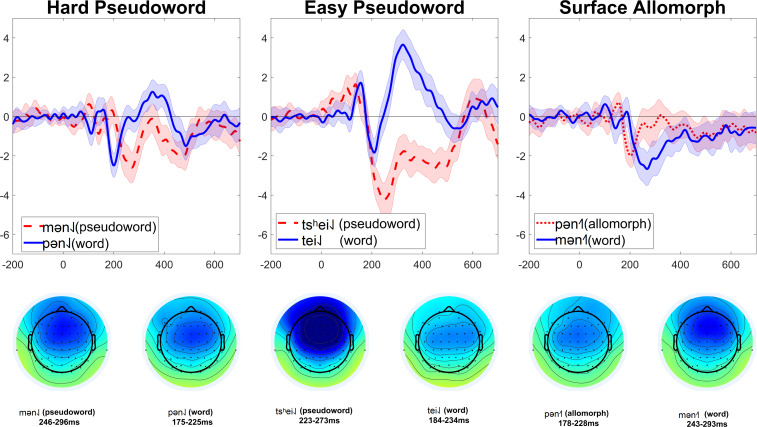
MMNs elicited in Experiment 2. Each subplot compares the MMN (at electrode Fz) elicited by one critical stimulus type (harder pseudowords, easier pseudowords, surface allomorphs) to that elicited by corresponding real-word stimuli (i.e., the subplot labeled “Hard pseudoword” compares the MMN elicited by pseudoword deviants to that elicited by real-word deviants). Each MMN is the result of subtracting ERPs elicited by a given stimulus in the control block from the ERPs elicited by the same stimulus presented as a deviant in an oddball block. The shaded ribbon around each MMN wave is a difference-adjusted Cousineau–Morey interval (Cousineau, 2005; Morey, 2008); at any given time point, if one MMN’s interval does not include the other MMN’s mean and vice versa, the two MMNs are likely to be significantly different from one another at that time point. The bottom portion of the figure shows topographic plots of each MMN from a 51-ms time window centered on that MMN’s most negative point.

**FIGURE 4:**
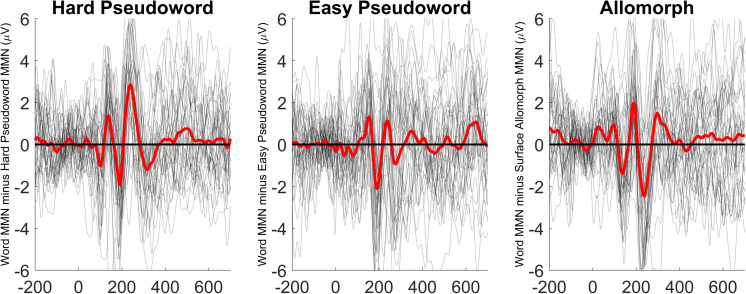
Lexicality effects (MMN elicited by word deviants minus MMN elicited by the corresponding pseudoword or surface allomorph deviants) for each participant (gray lines) as well as the grand average of these effects (thick red line), at electrode Fz.

The lowest number of trials remaining in a single cell was 55 (37%). MMNs (at electrode Fz) for each condition are shown in Figure 3. Lexicality difference waves (real-word MMN minus pseudoword or surface-allomorph MMN) for each participant are shown in Figure 4. Supplementary Figures showing ERPs at all scalp channels are available at https://osf.io/f4rnw/. Significant or marginal MMNs (i.e., significantly more negative ERPs for deviants than for corresponding control-block stimuli) were observed in every condition (see Table 3).

**TABLE 3:** Summary of Experiment 2 results.

Comparison	Real-word deviant	Other deviant	MMN for real-word deviant	MMN for other deviant	Real word MMN vs. other MMN
Hard pseudoword vs. real word	[pən˨˩]	[mən˨˩]	423–600 ms, *p* = 0.003	375–525 ms, *p* = 0.004	Real≈Pseudo, *p* = 0.533
Easy pseudoword vs. real word	[tei˨˩]	[tsʰei˨˩]	179–251 ms, *p* = 0.052	186–554 ms, *p* < 0.001	Real < Pseudo, *p* < 0.001
Allomorph vs. real word	[mən˧˥]	[pən˧˥]	202–570 ms, *p* < 0.001	415–565 ms, *p* = 0.011	Real≈Allomorph, *p* = 0.107

*The results shown here for the hard pseudoword row and the allomorph row are from the Exploratory Analysis, and the results for the easy pseudoword are from the initial analysis. “Other” refers to whichever condition is not the real-word condition in that comparison (e.g., in the comparison between real words and pseudowords, the “other” deviant is the pseudoword deviant). The columns “MMN for real-word deviant” and “MMN for other deviant” report the p-value for the one-tailed test comparing the deviant to the corresponding standard; the column “Real word MMN vs. other MMN” reports the p-value for the test (one-tailed for hard pseudoword and allomorph, two-tailed test for easy pseudoword) comparing the real-word deviant’s MMN to the other deviant’s MMN. The comparisons reported in this column are from the exploratory analysis reported below. Note that “Real < Pseudo” in the table indicates that the MMN for real words is smaller (i.e., more positive) than the MMN for pseudowords.*

The patterns are less clear in this experiment than they were in Experiment 1. In both the hard pseudoword comparison and the surface allomorph comparison (the two comparisons that replicate Experiment 1), there are some time windows where the MMN for real words is more negative than that for pseudowords or allomorphs, and some time windows where it is more positive. For instance, for the comparison between real words and harder pseudowords, real words appear to elicit a more negative MMN than pseudowords just before 200 ms; just after 200 ms, however, the pattern reverses. Or, to look at it another way: the MMN for harder pseudowords appears to peak earlier than the one for corresponding real words, whereas the MMN for surface allomorphs appears to peak later (and with a larger amplitude) than the one for corresponding real words.

It is apparent from these figures that real words did not reliably elicit more negative MMNs than uncontroversial pseudowords. Statistical analysis (summarized in Table 3) confirmed this impression.

In the manipulation-check comparison between real words and harder pseudowords, real words did in fact elicit more negative MMNs than hard pseudowords (*p* = 0.036), a difference that was driven by a broadly distributed cluster of 60 channels lasting from 175 to 235 ms after vowel onset. As the MMN waves for real words and pseudowords clearly crisscrossed over one another at various times, we ran an additional exploratory analysis using two-tailed tests (as opposed to the initial analysis with one-tailed tests, which only tested for the hypothesis that real words elicit more negative MMNs than pseudowords somewhere in the epoch). The two-tailed tests suggest that real words elicit both more negative MMN than pseudowords (*p* = 0.012, driven by a cluster of 60 channels from 177 to 233 ms) *and* more positive MMNs than pseudowords (*p* = 0.001, driven by a cluster of 48 channels from 233 to 467 ms). In other words, there is not a clear reliable trend for one condition to elicit robustly more negative MMNs than the other; the MMN elicited by real words is both more negative and more positive than pseudowords, depending on where one looks.

We repeated these analyses for the other comparisons as well. For real words vs. easier pseudowords, one-tailed tests revealed that the MMN for real words was not significantly more negative than that for pseudowords (*p* = 0.293). With two-tailed tests, the MMN for real words is still not significantly more negative than real words (*p* = 0.204) but is significantly more positive (*p* < 0.001, based on a cluster of 60 channels from 204 to 524 ms). Notice also that, as shown in Figure 3, the real-word stimuli in this comparison show a huge *positive* effect which appears to dwarf the MMN component itself; this pattern is not apparent in the easy pseudoword stimuli. This might be indicative that the real-word deviants elicited a much larger P300 than the easy pseudoword deviants; as the present experiment did not manipulate attention, there is not a straightforward way to disentangle overlapping MMN and P300 effects here. (Note, however, that this criticism applies equally to previous studies reporting modulation of MMN by lexicality; these experiments generally did not include manipulations designed to test whether these effects were due to real words eliciting larger MMNs or real words eliciting smaller P300s). In any case, we do not observe evidence that real words elicit a larger MMN than easy pseudowords.

While most of the effects reported here are qualitatively similar across different ways of calculating the MMN, the difference between real words and easier pseudowords was affected by the MMN calculation method. The difference reported above is based on the newer way of calculating MMNs, which in theory is better at isolating the MMN from the N1. When calculating the MMNs the older way (subtracting the standard from the deviant, as done in Experiment 1), we obtained a different pattern of results. These results are described in Supplementary File 6. In short, the results from that analysis show a similar criss-crossing pattern as that observed for the words and harder pseudowords reported above, but with the real words eliciting more negative (and not significantly more positive) MMN difference waves than easy pseudowords. Those results may provide some suggestive evidence that MMN is modulated by lexicality when the pseudowords are easy enough to recognize as pseudowords, but since that effect was not robust across different methods of calculating the MMN, it is not very convincing. For the rest of the paper we focus on the results from the newer way of calculating MMN, which we consider a preferable method for isolating the MMN from other nearby components.

As the predicted lexicality effect was not obtained with either harder or easier pseudowords, any differences between surface allomorphs and real words are not interpretable; nonetheless, we report these comparisons here for the sake of completeness. In this comparison, one-tailed tests revealed a significantly more negative ERP for real words than for surface allomorphs (*p* = 0.009, driven by a cluster of 58 channels from 227 to 361 ms). With two-tailed tests, the MMN for real words is both significantly more negative (*p* = 0.004, driven by more or less the same cluster of 57 channels from 228 to 353 ms) and significantly more positive (*p* = 0.040, driven by a cluster of 55 channels from 176 to 215 ms) than for surface allomorphs. In other words, the situation is the same as that for the comparison between words and hard pseudowords: the two waves cross back and forth over another, with neither being reliably more negative than the other.

### Exploratory Analysis

The results for the comparison between real words and harder pseudowords, as well as the comparison between real words and surface allomorphs, are indeterminate. In the initial, planned analysis, a cluster-based permutation test yielded a significant p-value indicating that the MMNs for words and non-words are different, but visual inspection of the waves suggests that this difference was not systematic. The MMNs crossed back and forth over one another, and thus the one-tailed cluster-based test may not have been an appropriate way to capture the difference, given that this test may have capitalized on the unsystematic spots where the real-word MMN was bigger than the pseudoword MMN while ignoring the spots where it was smaller. Two-tailed tests confirm that the MMN difference wave for real words was both bigger and smaller than that for pseudowords or surface allomorphs, depending on the time window examined. In a situation like this, where the data do not pass the inter-ocular trauma test (i.e., there is not a striking systematic difference between the two waves), we are hesitant to conclude that there is a robust difference; it would be more appropriate to conclude that the data are indeterminate and uninformative, consistent both with the presence and with the absence of a lexicality effect.

**FIGURE 5:**
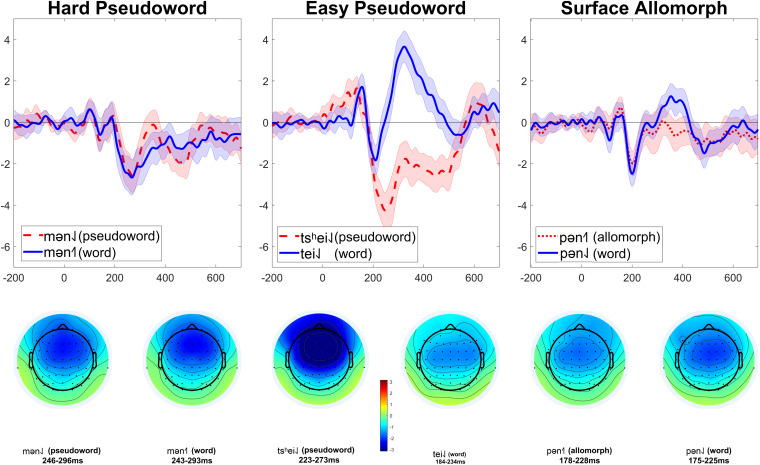
MMNs elicited in Experiment 2. These are the same data as in Figure 3, but comparing different combinations of conditions; see text for details.

Here, we report an additional exploratory analysis which provides more evidence against the presence of a lexicality effect in these conditions. For this analysis, we changed the conditions being compared, as shown in Figure 5. The harder pseudoword [mən˨˩] we now compare to the real word [mən˧˥] instead of the real word [pən˨˩]; in other words, we are now comparing the pseudoword to a word with the same onset but a different rime, rather than comparing it to a pseudoword with the same rime but a different onset. Likewise, the harder pseudoword [pən˧˥] we are now comparing to the real word [pən˨˩] instead of the real word [mən˧˥].

Why is the latter comparison more appropriate? In Experiment 1, we compared MMNs within the same rime and different onsets: real-word [kʰa˨˩] to pseudoword [pʰa˨˩], and real-word [pʰa˧˥] to surface allomorph [kʰa˧˥]. In that experiment, this seemed appropriate because we were concerned that words with different tones would elicit substantially different MMNs, particularly because low tone ([kʰa˨˩] and [pʰa˨˩]) is typically longer than rising tone ([kʰa˧˥] and [pʰa˧˥]). This was especially important in the context of Experiment 1, where the MMNs came from a contrast between standards that had diphthongs (such as [kʰai˨˩]) and deviants that had monophthongs (such as [kʰa˨˩]). Since syllables with low tone are longer than syllables with rising tone, and the disambiguation point between the diphthong and monophthong will come somewhere in the middle of the vowel, it was likely that the recognition point at which participants realize they are hearing a monophthong deviant would be later, and perhaps more variable, in the low-tone stimuli than the rising-tone stimuli. Consequently, the peak of the MMN in the low-tone stimuli does appear to be later in the low-tone deviants (left side of Figure 1) than in the rising-tone deviants (right side of Figure 1), and the morphology of the MMN seems messier. For these reasons, we considered this comparison to be the more appropriate one. While it necessitated comparing MMNs elicited by deviants with different onset consonants ([kʰ] vs. [pʰ]), we considered this to be a minor concern because (a) [kʰ] and [pʰ] are acoustically fairly similar, and (b) differences in the endogenous ERP response to [kʰ] and [pʰ] should be subtracted out of the MMNs due to the subtraction method, given that responses to [kʰa] deviants were subtracted from responses to [kʰai] standards, and [pʰa] deviants from [pʰai] standards.

For Experiment 2, we followed the same approach, but in retrospect this comparison was probably not appropriate this time. Firstly, in Experiment 2 this required comparing MMNs elicited by deviants with different onsets [p] vs. [m], and the acoustic difference between [p] and [m] is far larger than that between [k^h^] and [p^h^]. Secondly, the standard/deviant contrast used to elicit MMNs was different in this experiment: participants had to detect the transition from e.g., [pan˨˩] standards to [pən˨˩] deviants. This contrast was intentionally chosen to make the standard-deviant difference noticeable at the vowel onset, instead of only being noticeable at some point well into the vowel as it was in Experiment 1, which used a diphthong-monophthong contrast. Since Experiment 2 used a contrast which should be easily detectable right at the vowel onset, the intrinsic duration difference between low- and rising-tone syllables is less likely to influence the MMN in the way it did in Experiment 1. On the other hand, since [p] and [m] onsets are so physically different, especially in terms of the nature of their transition into the following vowel, these may have had substantial influence on the detection of the standard-deviant transition, and thus on the morphology of the MMN. That might be the reason why, as can be seen in Figures 3, 5, the MMNs for [p]-initial and [m]-initial deviants with the same tones crisscross over one another when they are plotted together (Figure 3), whereas the MMNs for low- and rising-tone deviants with the same onsets overlap nicely (Figure 5). While all this reasoning is *post hoc*, we believe it justifies our use of different comparisons in Experiment 1 (comparing MMNs elicited by deviants with the same rimes but different onsets) vs. Experiment 2 (comparing MMNs elicited by deviants with the same onsets but different rimes).

This exploratory analysis suggests that there was not a lexicality effect in these conditions. While the initial planned analysis compared pseudowords or surface allomorphs to real words that had the same rime and a different onset (e.g., real-word [pən˨˩] vs. pseudoword [mən˨˩], and real-word [mən˧˥] vs. surface allomorph [pən˧˥]), these turned out to be a poor comparison, since the MMNs for words with different onsets were clearly more different than the MMNs for words with different rimes. When we re-analyzed the data by comparing MMNs within the same onset (e.g., real-word [pən˨˩] vs. pseudoword [pən˧˥], and real-word [mən˧˥] vs. surface allomorph [mən˨˩]), there was no significant lexicality effect. For the comparison between real words and harder pseudowords, real words did not elicit significantly more negative MMNs than pseudowords (*p* = 0.533). For the comparison between real words and surface allomorphs, real words again did not elicit significantly more negative difference waves (*p* = 0.107).

### Discussion

While the results of Experiment 2 are less straightforward than those of Experiment 1, the experiments taken together provide fairly strong evidence that there was not a lexicality effect when we compared real words to the most word-like pseudowords—pseudowords that differ from real words only by tone, and which are harder to recognize as pseudowords. Less word-like pseudowords (those that differ from real words by at least one segment, and are easier to recognize as pseudowords) also failed to elicit weaker MMNs than real words (see, however, Supplementary File 6 for an alternative, sub-optimal analysis which did observe a weak lexicality effect in the expected direction). Thus, the results suggest that lexicality effects either do not occur at all, or only occur with certain kinds of pseudowords.

## General Discussion

Across two experiments, we found fairly convincing evidence that the most word-like pseudowords (pseudowords which have the same segments but different tone than a real word) did not elicit significantly smaller MMNs than real words. In other words, we failed to observe the expected lexicality effect. Here, we consider several possible explanations for why this happened.

### Type II Error

When a single experiment fails to observe an effect, it is of course impossible to rule out the possibility that this result represents a type II error (i.e., a false acceptance of the null hypothesis of no effect, when in reality a non-zero effect does exist in the population). We also cannot rule out this possibility, although we think it is unlikely. First of all, our experiments were designed to have high power. While the estimation of power in ERP experiments is difficult, as it depends not just on sample size and effect size but also on number of trials and on multiple variance components that are difficult to estimate (e.g., variance between trials within a condition within a participant), we nevertheless had the largest number of participants out of any studies on the MMN lexicality effect reported thus far, and we have no reason to believe that other aspects of our design would have reduced power enough to counteract this. Furthermore, we replicated this lack of lexicality effect across two experiments (although the results of the second experiment are, admittedly, less clear-cut than those of the first experiment).

### Unknown Confound in the Stimuli

This, of course, is another factor that cannot be ruled out. While we did our best to ensure that the pseudoword and real-word stimuli were as similar as possible and did not differ in any important ways other than their lexical status, it is always possible that there is some confounding factor we failed to consider. This would not explain our results, however, unless such a confound could actually be identified.

### Homophony

One possible such confound we have considered has been homophony – many Mandarin syllables correspond to multiple morphemes. The traditional explanation for why real words elicit a larger MMN than non-words, per Pulvermüller et al. (2001), is that hearing a sound and activating its associated meaning triggers the activation of a cortical cell assembly, whereas hearing a sound without any associated meaning does not do so. Is it possible that this extra activation does not happen when a listener cannot uniquely identify one lexical entry to activate when they hear a sound? This is an interesting possibility to investigate, but it does not seem likely to explain our results. In Experiment 1, the real word in the manipulation check was [kʰa˨˩], which only corresponds to one morpheme. In Experiment 2, the real word in the manipulation check (in the exploratory analysis) was [mən˧˥n], which corresponds to two morphemes (門 “door,” and a plural inflectional morpheme 們); but even if this issue explains the lack of lexicality effect for that comparison in Experiment 2, it would not explain the concomitant lack of lexicality effect in Experiment 1.

### Neighborhood Density

Another situation where lexicality effects occur is the prime lexicality effect in priming experiments (e.g., Forster and Veres, 1998; Qiao et al., 2009). In primed lexical decision tasks with visually presented targets and primes, visible primes that share a phonological or orthographic relation with the targets facilitate reaction times to the target when the primes are pseudowords, but not when they are real words. This effect, however, seems to only occur when the primes have few neighbors—e.g., for primes like UNIVORSE (Forster and Veres, 1998). In Mandarin, the sorts of single-syllable stimuli used here certainly all have dense phonological neighborhoods. However, the prime lexicality effect comes from a much different paradigm than the MMN, and it is not clear if the conclusions from that paradigm also apply here. It is possible that the neighborhood density effect in those studies does not occur because lexicality effects themselves are modulated by neighborhood density, but instead because priming (on which the lexicality effects in those studies depend) is modulated by neighborhood density. Furthermore, lexicality effects on the MMN in other languages have been observed with monosyllabic stimuli from fairly dense phonological neighborhoods (e.g., *tight* vs. *bite* in Shtyrov and Pulvermüller, 2002). Thus, we do not think the high neighborhood density of our stimuli would necessarily be expected to preclude them from showing lexicality effects in an MMN paradigm.

### Lexicality of Standards

As discussed above in our description of the stimuli, we designed the experiment around the lexicality of the deviants. While we tried to match the lexicality of the standards as much as possible, it was not our primary concern, given that the results from Shtyrov and Pulvermüller (2002) suggest that lexical status of standards does not influence the MMN. We cannot, however, rule out the possibility that *those* results were a Type II error. As it is now, assuming that our results cannot be explained by confounds related to lexical status of the standards requires accepting the conclusions of an almost 20-year-old study with only 10 participants. While we see no particular reason to doubt the validity of that study (the sample size is small, but is representative of MMN studies from that time), it does seem reasonable, from an Occam’s Razor perspective, that we should first question whether lexicality of standards really does not matter before we question whether the MMN lexicality effect exists at all, given that the former assumption is based on one study whereas the latter assumption is based on many. Thus, the issue of the lexicality of standard stimuli probably deserves further research and replication; we cannot rule it out at this time. For future research aiming to better understand whether (or how) lexicality modulates the MMN, addressing the question of lexicality of the standards should be one of the top priorities.

### Mandarin Is Special

Is there some reason that lexicality effects which may be robust in other languages do not occur in Mandarin? We cannot rule this out. However, we do not consider this an acceptable explanation for the present results unless a specific, testable, and falsifiable mechanism for this special status of Mandarin is presented.

### The Lexicality Effect Doesn’t Occur When Pseudowords Are too Similar to Real Words

This is essentially a more specific version of the possibility raised immediately above – rather than just assuming that there is some nebulous special property of Mandarin, here we are focusing on a specific special property of the Mandarin pseudowords we used. This account seems to be the most consistent with our results, given that we found fairly strong evidence that the lexicality effect does not occur in the most word-like pseudowords but found some weak evidence that it might occur in less word-like pseudowords. *Why* this lexicality effect would depend on the wordlikeness of pseudowords, however, is an open question. This claim seems to assume that the lexicality effect is rooted in at least somewhat attentional mechanisms, if words that are difficult to identify as pseudowords do not reduce the MMN amplitude; such a conclusion, however, would be in conflict with what we know about the MMN, which is that the MMN is generated by pre-attentive processes. Perhaps the so-called MMN lexicality effect is not a modulation of the MMN at all, but modulation of some other component that overlaps with it, such as the P300. Alternatively, maybe the difference between pseudoword types does not depend on attention. It remains unclear, though, why less word-like pseudowords would behave differently than more word-like pseudowords in this paradigm. Recall that Pulvermüller et al. (2001) proposed explanation for the lexicality effect is that hearing real words triggers the activation of a cortical cell assembly and hearing pseudowords does not. Under such an account, why would it matter how word-like the pseudoword is? One possibility is that a person perceiving these sounds is doing some kind of fuzzy matching: maybe a pseudoword that is too similar to a real word is sufficient to accidentally activate cortical assemblies for real words anyway. This possibility is worth further study. Indeed there is a sizeable literature on priming and lexical activation in situations where stimuli almost match lexical representations, as well as a sizeable literature on the role of tone in Mandarin lexical activation. This literature may offer some guidance for the present question. However, it does not seem wholly consistent with the speculation about fuzzy matching that we have sketched above. Our speculation would require assuming that the lexical recognition device can ignore tone enough to activate lexical entries which share segments but not tone with the input; priming studies on tone in Mandarin, however, show that tone is not wholly ignored (e.g., Sereno and Lee, 2015): input with one tone does somewhat activate lexical entries with other tones, but not as much as input with the correct tone does.

### The Lexicality Effect on the MMN Is Not Real or Not Replicable

The final possibility is that the lexicality effect is not a reliable effect. This is indeed suggested by the summary of studies in Table 1: most recent MMN studies comparing words and non-words did not observe the effect. The mechanisms by which such an effect would occur are also unclear, as the cell-assembly idea which Pulvermüller et al. (2001) have used to explain it has been heavily challenged on linguistic grounds (see, e.g., the commentaries on Pulvermüller, 1999, many of which point out that this mechanism may not work for abstract words and function words). Nevertheless, we do not believe our results necessarily support such a drastic conclusion. First of all, as mentioned above, rather than assuming that the lexicality effect itself is not real, another possibility is that some of the less tested assumptions on which our experiments relied (particularly, the assumption that the lexicality of the standards does not need to be controlled) were faulty. Secondly, our supplementary analyses offer suggestive evidence that there may be at least one hidden moderator behind the lexicality effect: it might happen when real words are compared to less word-like pseudowords but not to more word-like pseudowords. While we do not find this possibility convincing (given that it relies on the results of a sub-optimal analysis technique), we must acknowledge that it is a possibility which has yet to be ruled out. It is possible that this, or other moderators like it, might explain why, as reviewed in Table 1, some studies observe a lexicality effect and some studies do not.

In short, our experiments failed to observe a lexicality effect with more word-like pseudowords, and did not convincingly observe such an effect for less word-like pseudowords either. These results suggest that our understanding of what may or may not cause real words and pseudowords to elicit different MMN patterns is still limited. Better understanding this question will have important implications for studies using the MMN to address psycholinguistic and linguistic questions, because in order to effectively deploy such paradigms we will need to have a clear understanding of what properties of the stimuli modulate the MMN and what properties do not. The present study highlights the need for more research into this question, as far as lexical status of MMN stimuli is concerned.

## Data Availability Statement

The datasets presented in this study can be found in online repositories. The names of the repository/repositories and accession number(s) can be found below: https://osf.io/f4rnw/.

## Ethics Statement

The studies involving human participants were reviewed and approved by Human Subjects Ethics Sub-committee at the Hong Kong Polytechnic University. The patients/participants provided their written informed consent to participate in this study.

## Author Contributions

SP-A conceptualized the experiment. SI prepared the stimuli. SP-A prepared the experiment protocol. SI collected the data. SI and SP-A analyzed the data. SP-A wrote the first draft of the manuscript. SP-A and SI gave final approval for publication. Both authors contributed to the article and approved the submitted version.

## Conflict of Interest

The authors declare that the research was conducted in the absence of any commercial or financial relationships that could be construed as a potential conflict of interest.

## Footnotes

1 Here we refer to these as “surface allomorphs” for simplicity, given the fact that they exist *only* as surface allomorphs and not as citation forms; of course, technically speaking, all of the stimuli used in this experiment are surface allomorphs of something, but [kʰa˨˩] is the only one that exists *only* as a surface allomorph and not as an underlying form.

2 https://politzerahles.shinyapps.io/ERPpowersim/
