# Supplementary material for: Mismatch Negativity Is Not Always Modulated by Lexicality
Source: Front Hum Neurosci. 2020 Oct 30;14:556457. doi: 10.3389/fnhum.2020.556457 (PMC7662093; doi:10.3389/fnhum.2020.556457)
Supplement: Supplementary file 1 [file Presentation_6.pdf]

Supplementary File 6. Results for the Word – Easy Pseudoword comparison based on traditional MMN derivation

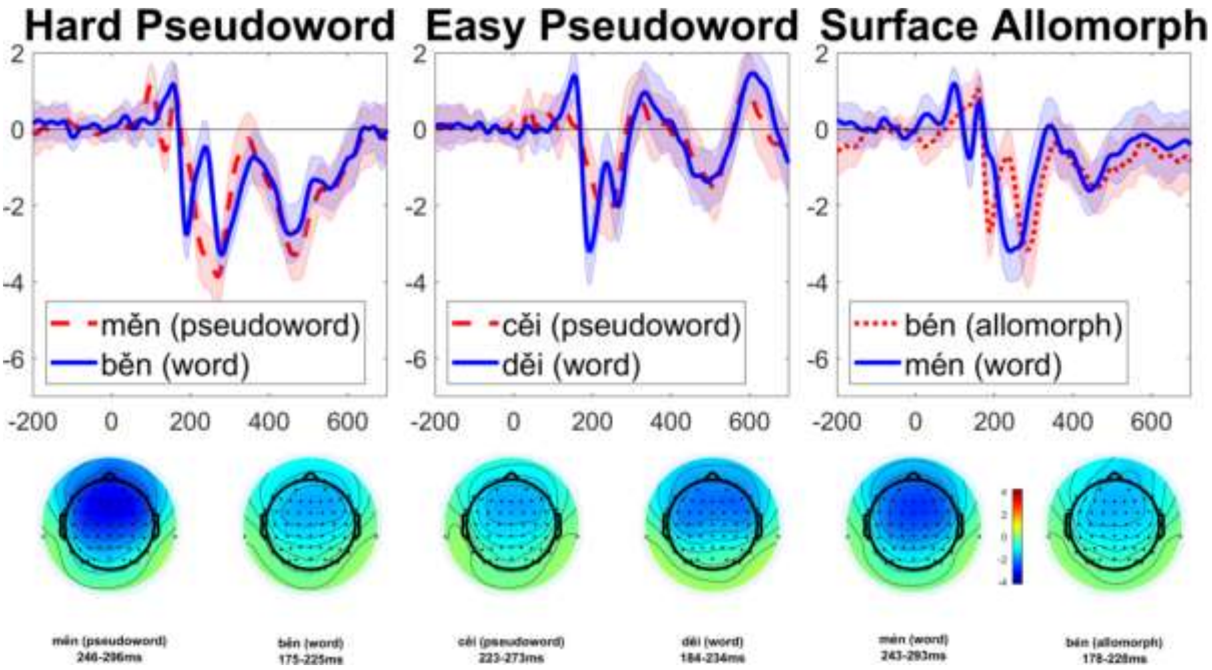

The figure above shows the MMNs (following the same format as Figure 3 in the main paper). The results for the Word – Hard Pseudoword comparison (left panel) and Word – Surface Allomorph comparison (right panel) are qualitatively similar to the results reported in the main text and thus will not be discussed further here. However, the results for the Word – Easy Pseudoword comparison (center panel) are quite different. In the analysis reported in the main text, words elicited a substantially more *positive* MMN difference wave (driven by what appears to be a massive P300 effect) than easy pseudowords. In the figure shown above, however, the large positivity is absent, and words and easy pseudowords elicit qualitatively similar patterns, with the two waves criss-crossing over one another. The below figure shows this comparison at every electrode in the montage.

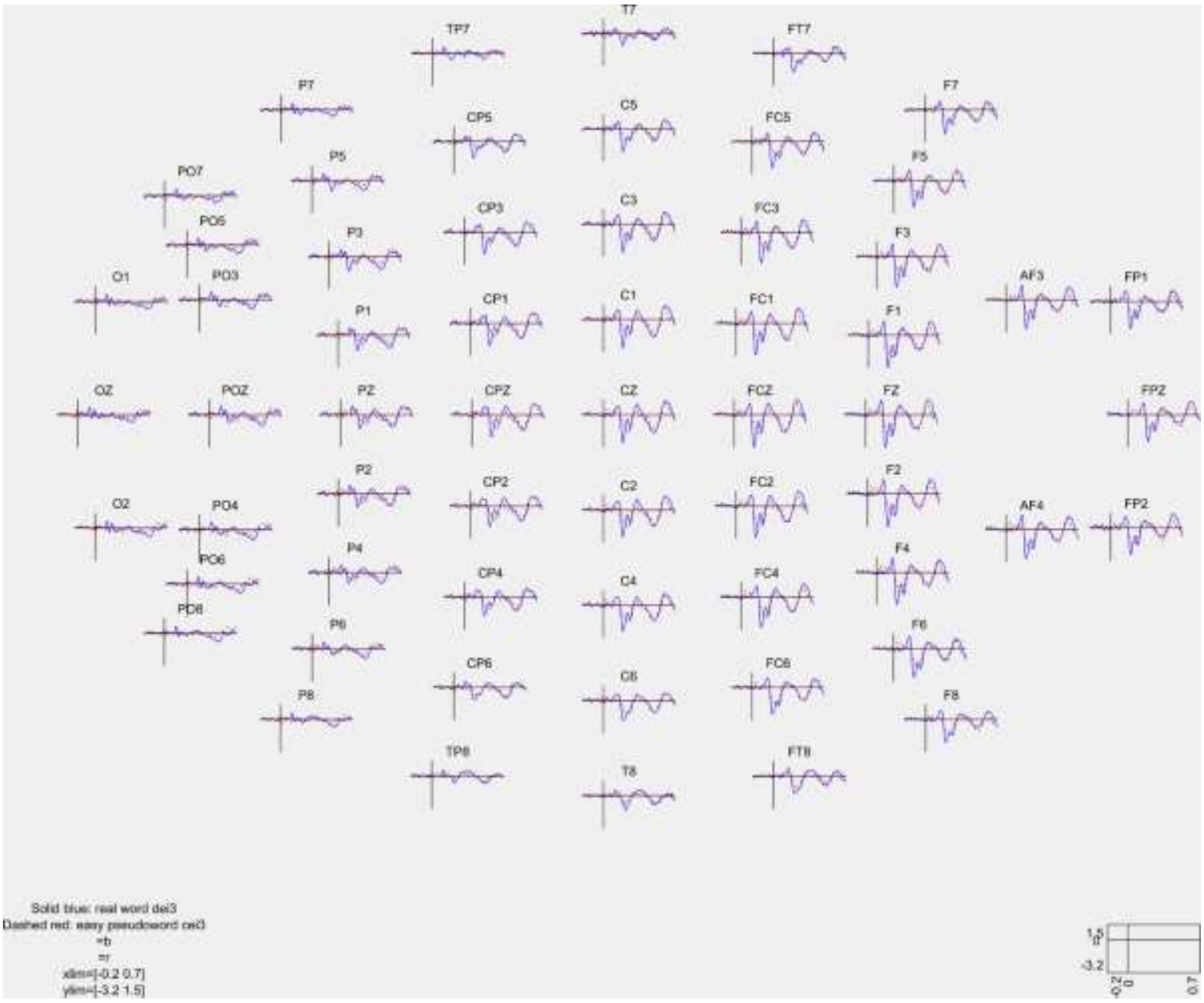

In the one-tailed cluster test, real words elicited marginally more negative MMNs than easy pseudowords (*p*=.069, based on a cluster of 52 channels from 168 to 219 ms). In the two-tailed cluster test, real words elicited a significantly more negative MMN than easy pseudowords (*p*=.034, based on a cluster of 50 channels from 169 to 216 ms) and did not elicit significantly more positive MMNs (*p*=.125).

These results suggest that real words may have elicited slightly larger MMNs than easy pseudowords, when the MMN is calculated by subtracting the standards (elicited by different physical stimuli than the deviants) from the deviants in the same block. However, this effect did not hold when calculating MMNs based on a control block, as described in the main paper. As that method is arguably better for identifying MMNs, as it subtracts out N1 activity that may otherwise be present in the MMN difference wave, we consider the results from that method more convincing than the results from this method.

Direct comparison between lexicality effects

The above analysis provides some weak evidence that real words may elicit larger MMNs than easier (less word-like) pseudowords. Furthermore, real words

do not elicit larger MMNs than harder (more word-like) pseudowords. Nevertheless, these two observations are not yet evidence that the lexicality effect with easier pseudowords is stronger than the lexicality effect with harder pseudowords. Showing a significant effect in one comparison and not in another comparison is not sufficient to prove that the two comparisons are significantly different from one another; an interaction test is necessary to do that (Gelman & Stern, 2006). Here we report a direct comparison of the lexicality effect with easier pseudowords to the lexicality effect with harder pseudowords, i.e., a difference-of-difference-of-differences tests: { (real-word MMN minus easier pseudoword MMN) minus (real-word MMN minus harder pseudoword MMN) }. Here we are quantifying the harder-pseudoword lexicality effect using the exploratory comparison between deviants with the same onsets (see the Exploratory Analysis reported in the main text), and in both cases we are analyzing MMNs based on subtracting the standard from the deviant.

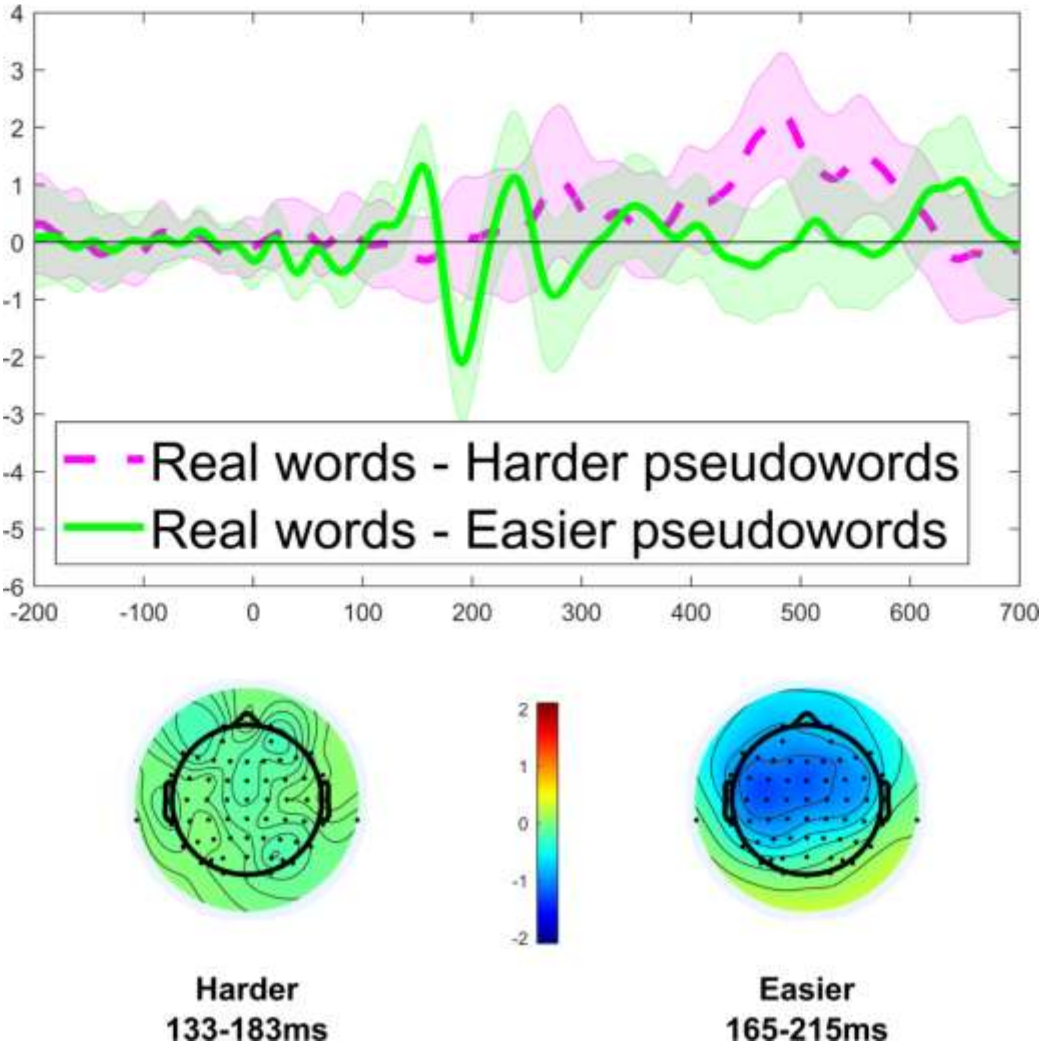

The two lexicality effects (i.e., difference-of-difference waves) are shown in the figure above. The figure shows that real words elicited a substantially more negative MMN than easier pseudowords around 200 ms after vowel onset, whereas no such lexicality effect appeared in the comparison with harder

pseudowords. More importantly, the difference wave with easier pseudowords appears to be significantly more negative than the difference wave with harder pseudowords at around 200 ms (and again at around 300 ms). A spatiotemporal cluster-based test confirmed this observation. The test followed the same parameters as the previous tests, except that the window of analysis only went up to 400 ms, as we wanted to exclude the late portion of the time window in which the lexicality effects were different just because the harder-pseudoword comparison elicited a lexicality effect in the opposite direction we expected (a more positive difference wave for pseudowords than for real words), for which we have no interpretation (this may be reflecting P300 activity rather than MMN activity). The test showed that the difference-of-differences wave for the easier-pseudoword lexicality effect was marginally more negative than the difference-of-differences wave for the harder-pseudoword lexicality effect (p=.056), an effect which was driven by a widely distributed, slightly more frontal than posterior, cluster of 49 channels lasting from 169 to 216 ms.

While these results are necessarily tentative, because the easier-pseudoword comparison was calculated in a different way than the harder-pseudoword comparison, they provide some preliminary evidence that the lexicality effect might be modulated by the type of pseudoword being used.

References

Gelman, A., & Stern, H. (2006). The difference between "significant" and "not significant" is not itself statistically significant. *The American Statistician, 60*, 328-331.
